# oxLDL inhibits differentiation and functional activity of osteoclasts via scavenger receptor-A mediated autophagy and cathepsin K secretion

**DOI:** 10.1038/s41598-018-29963-w

**Published:** 2018-08-02

**Authors:** Damilola Dawodu, Margret Patecki, Jan Hegermann, Inna Dumler, Hermann Haller, Yulia Kiyan

**Affiliations:** 1 Department of Nephrology and Hypertensiology, Hannover Medical School, Hannover, Germany; 2 Research Core Unit Electron Microscopy, Hannover Medical School, Hannover, Germany

## Abstract

Resorptive activity of osteoclasts is important for maintaining bone homeostasis. Endogenous compounds such as oxidized low density lipoprotein (oxLDL) have been shown to disturb this activity. While some studies have investigated the effects of oxLDL on the process of osteoclastogenesis, the underlying mechanism are not fully understood. We show here that oxLDL concentrations of ~10–25 µg protein (0.43–1.0 µM MDA/mg protein) completely blocked the formation of functional osteoclasts. The underlying mechanism implies an inhibition of autophagy that in turn leads to a decreased fusion of cathepsin K (CatK)-loaded lysosomal vesicles with the ruffled border membrane. As result, a lower secretion of CatK and impaired protonation of the resorption lacunae by vacuolar-ATPase (v-ATPase) is observed in the presence of oxLDL. We demonstrate that scavenger receptor A (SR-A) mediates oxLDL effects on osteoclastogenesis and repressing this receptor partially rescued oxLDL effects. Collectively, our data provides an insight into the possible mechanism of oxLDL on osteoclastogenesis suggesting that it does not perturb the packaging of CatK and v-ATPase (V-a3) in the secretory lysosome, but inhibits the fusion of these lysosomes to the ruffled border. The relevance of our findings suggests a distinct link between oxLDL, autophagy and osteoclastogenesis.

## Introduction

Cardiovascular disease (CVD) is the most common cause of mortality in the general population. While several factors such as uremia, inflammation, vascular calcification etc. have been shown to contribute to CVD^1^, studies have shown that total and LDL-cholesterol are the two most independent risk factors of cardiovascular morbidity and mortality^2^. LDL is oxidatively modified *in vivo* into either minimally modified LDL (mmLDL) or extensively oxidized LDL (oxLDL), increasing its proinflammatory and proatherogenic properties^3^. mmLDL differs from oxLDL in that it still binds to the LDL receptor while oxLDL interacts with scavenger receptors such as SR-A, TLR-4, CD36 and LOX-1 leading to a signaling cascade that induces foam cell formation, inflammation and plaque formation in the vessel walls^4^. Cardiovascular complications are also associated with mineral bone disorder^5^. Studies show an inverse relationship between bone mineral density and the prevalence for CVD in both men and women^5^. There is evidence that ossification of bone and vascular calcification of the vessel walls are modulated by osteoblasts and osteoclasts; the two cell types involved in bone remodeling. The intercellular communications between these two cell types must be tightly regulated to maintain bone homeostasis^6^, since an increased osteoblast activity would lead to osteopetrosis in the bone and calcification in the vessel walls while an increased osteoclast activity leads to diseases such as osteoporosis or inflammatory arthritis^7^. Osteoclasts are multinucleated cells derived from the monocyte/macrophage lineage and developed by the cell-cell fusion. The fusion of these cells are mediated by soluble factors produced by osteoblasts such as the macrophage colony-stimulating factor (M-CSF) and receptor activator of NF-κB ligand (RANKL)^8,9^. M-CSF is important for the proliferation and survival of the cells while RANKL is necessary for the differentiation to osteoclasts by inducing the fusion of mononuclear cells and initiating the expression of osteoclast specific genes such as cathepsin K (CatK)^10^. Functional activity of osteoclasts depends on the tight adhesion to the bone matrix mediated by vitronectin receptors, the formation of actin rings important for the development of sealing zones, the dissolution of inorganic bone mediated by the action of proton pumps, and secretion of lysosomal proteases crucial for organic bone resorption^11^.

Studies have shown that retention of endogenous compounds such as oxLDL can disturb the delicate balance between bone forming cells and bone resorbing cells thereby affecting bone homeostasis and bone remodeling^6,12–17^. We demonstrate that oxLDL inhibits the process of osteoclastogenesis and the functional activities of osteoclasts by disabling the expression and more importantly, the secretion of CatK through autophagy regulation.

## Results

### oxLDL is a potent inhibitor of osteoclastogenesis

**Figure 1:**
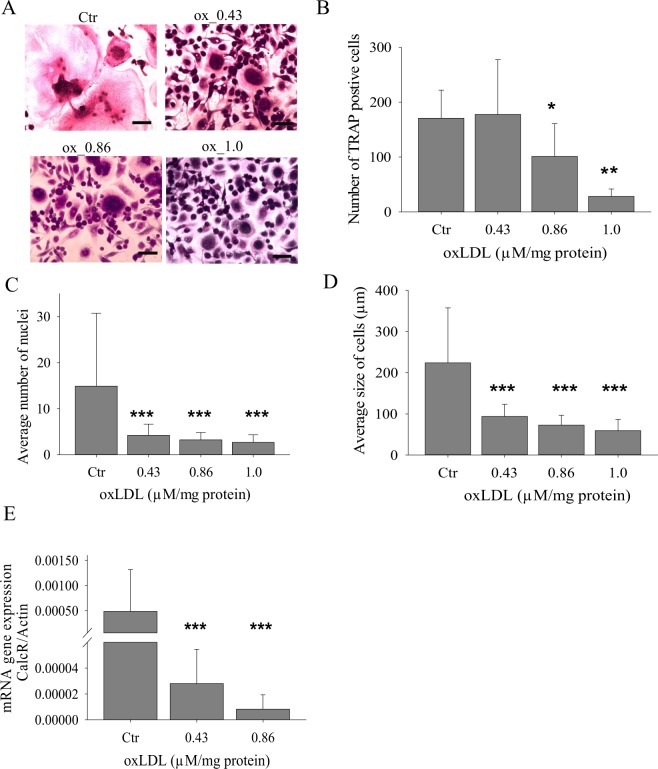
oxLDL inhibits fusion of mononuclear cells. Monocytes plated on glass coverslips were grown in the presence of M-CSF and RANKL for a total of 15 days, and then TRAP staining was carried out. (**A**) TRAP staining of control and oxLDL treated cells. (**B**) Quantification of the average number of TRAP positive cells. (**C**) Average number of nuclei per TRAP positive cell. (**D**) Quantification of the average size of TRAP positive cell. Magnification x25, scale bar 200 µm. n ≥ 3 (**E**) mRNA expression of CalcR with and without oxLDL.

Because osteoclasts become apoptotic after each resorptive cycle^11^, we were more interested in the effects of oxLDL on the process of osteoclastogenesis than on its activity of already formed osteoclasts. As shown by the TRAP staining (Fig. 1A), oxLDL inhibited RANKL-induced differentiation of macrophages to osteoclasts. oxLDL reduced the number of TRAP positive cells significantly at 0.86 µM and 1 µM oxLDL concentrations but not at 0.43 µM (Fig. 1B). The number of nuclei per TRAP positive cell was also significantly lower in the presence of oxLDL (Fig. 1C): ~3–6 nuclei per cell whereas a range of ~20–60 nuclei per cell in control group. oxLDL treated cells also had a 3 fold decrease in the average cell surface area compared with the control (Fig. 1D). Expression of calcitonin receptor, a marker for osteoclast differentiation, was also dose dependent downregulated in oxLDL treated cells confirming that oxLDL inhibits the formation and maturation of osteoclasts (Fig. 1E).

### oxLDL interferes with cytoskeletal rearrangement of osteoclasts

**Figure 2:**
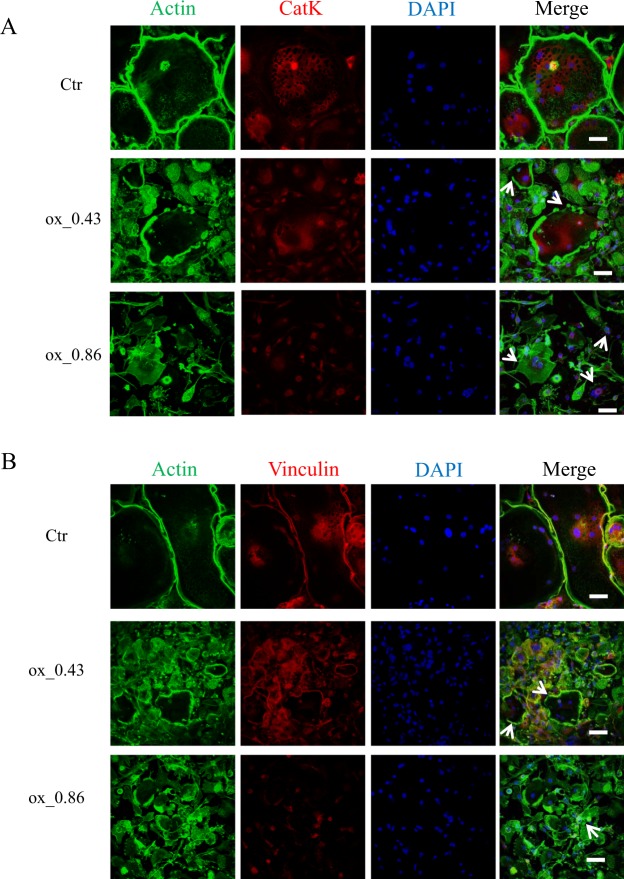
oxLDL inhibits the cytoskeletal rearrangement of osteoclasts. Monocytes plated on glass coverslips were grown in the presence of M-CSF and RANKL for a total of 15 days and cells were analyzed by confocal microscopy. (**A**) Mature osteoclasts were stained FITC-phalloidin (green) and with antibody against CatK (red) and nuclei (DAPI, blue). White arrows indicate disrupted cytoskeleton. (**B**) Mature osteoclasts were stained FITC-phalloidin (green) and with antibody against vinculin (red) and nuclei (DAPI, blue). White arrows indicate disrupted actin binding. Magnification x40, scale bar 50 µm. n ≥ 3.

During the differentiation of macrophages to osteoclasts, a reorganization of the cytoskeleton takes place resulting in the formation of actin rings. These structures are important for cell to matrix adhesion and the generation of sealing zones necessary for bone resorption^11^. We investigated the effects of oxLDL on the morphology of osteoclasts by performing immunocytochemical staining with phalloidin. As shown in Fig. 2A, control cells developed actin rings on the entire periphery of the cell with numerous nuclei (DAPI). However, oxLDL interfered with the cytoskeletal rearrangement at all oxLDL concentrations tested, leading to deformed actin ring structures focused on one side of the cell instead of the entire cell periphery. To further confirm the disruption of the cytoskeleton by oxLDL, vinculin, an actin binding protein was co-stained with actin. Confocal images showed vinculin co-localizing with actin rings on the entire periphery of control cells while in oxLDL treated cells, vinculin co-localization with actin rings was strongly diminished or absent (Fig. 2B).

In order to explain the possible cause of the cytoskeletal disruption by oxLDL, we checked the expression of the vitronectin receptor (αVβ3) which is known to mediate the tight adhesion of the cells to the bone matrix as the first step in the formation of actin rings^18^. We found that the expression of vitronectin receptor was not significantly different between control and oxLDL treated cells (Supplementary Fig. 1) suggesting a direct effect of oxLDL on the cytoskeletal rearrangement in osteoclasts.

### oxLDL completely inhibits resorption on hydroxyapatite coated plates and bone slices

**Figure 3:**
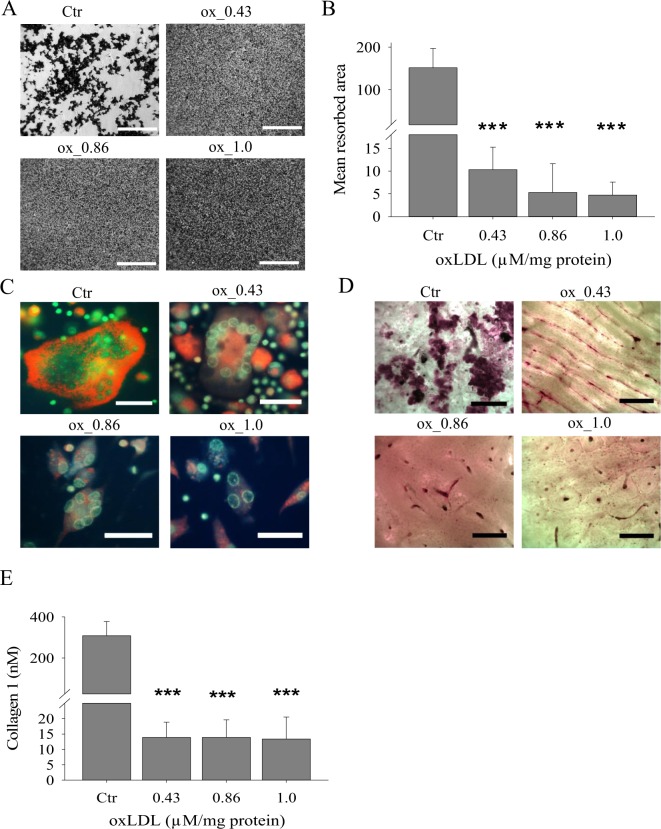
oxLDL blocks the functional activities of osteoclasts. Monocytes were plated on hydroxyapatite coated plates and bone slices for a total of 21 days. (**A**) Hydroxyapatite coated plates showing a total resorptive inhibition in the presence of oxLDL. Magnification x10, scale bar 1 mm. (**B**) Quantification of resorbed areas on hydroxyapatite coated plates with ImageJ. (**C**) Fluorescence microscopy of acridine orange staining of mature osteoclasts cultured on tissue culture plates showing v-ATPase mediated acidification. Green and orange indicate neutral and acidic pH respectively. Magnification x40, scale bar 1 mm (**D**) oxLDL inhibits resorptive activity of osteoclasts on bone slices. Magnification x10, scale bar 1.5 mm. (**E**) Conditioned media from D was collected and analyzed for the presence of collagen type 1 fragment using a colorimetric ELISA method. n ≥ 3.

A functional characteristic of osteoclast is the dissolution of inorganic bone components and resorption of organic bone matrix. We tested the effects of oxLDL by assessing the size of resorbed area. Cells were plated and differentiated to osteoclasts by addition of RANKL on both hydroxyapatite coated plates and bone slices, and cultured for a total of 21 days. Hydroxyapatite coated plates have been shown to provide a stable calcium phosphate layer that can be used reliably to quantify osteoclast resorption *in vitro*^11^. Treatment of these cells with oxLDL at all concentrations completely inhibited the dissolution of calcium crystals (Fig. 3A).

Next, we checked the protonation of acridine orange and observed that there was a higher protonated acridine orange (indicative of acidic organelles) in control cells and a dose dependent decrease in oxLDL treated cells (Fig. 3C). These acidic organelles were mainly localized at the periphery of control cells while in oxLDL treated cells they were confined to the cytoplasm suggesting that they have less capacity to acidify the resorption lacunae. Moreover, as visualized with 1% toluidine blue, oxLDL caused a total inhibition of bone resorption. Contrary to the control cells which had numerous and well defined pits, oxLDL at all concentrations completely impaired bone resorption, as ascertained by abrogated resorption pits and collagen type 1 fragment release into the medium (Fig. 3D,E).

### oxLDL inhibits the expression, activity and secretion of CatK and MMP9

**Figure 4:**
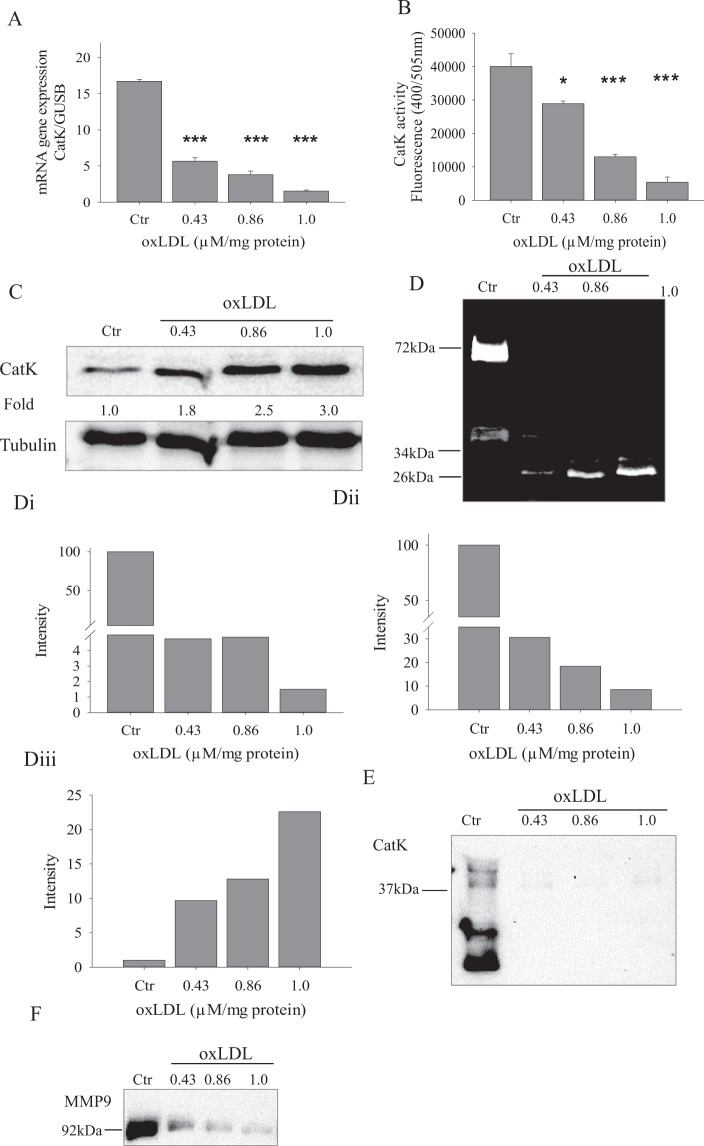
oxLDL regulates the gene expression and secretion of CatK. Osteoclasts were stimulated with different concentrations of oxLDL. (**A**) mRNA expression of CatK with and without oxLDL (**B**) CatK activity by fluorometric assay using synthetic substrate in control and oxLDL treated cells. (**C**) Cells treated with and without oxLDL for 3 days were lysed and equal amount of cell lysates were western blotted for CatK. (**D**) Conditioned media from control and oxLDL treated osteoclasts were subjected to gelatin zymography at day 8; intensity quantification in Di–iii. (**E**) Conditioned media were subjected to western blot analysis at day 8 and probed with the CatK antibody. (**F**) Conditioned media were subjected to western blot analysis at day 8 and probed with the MMP-9 antibody. Images from both zymography and western blot were captured using the VersaDoc‐3000 (Bio‐Rad Laboratories, Muenchen, Germany) and quantified using QuantityOne software (Bio‐Rad Laboratories). Error bars represent standard deviation. n ≥ 3.

CatK is an osteoclast specific protease responsible for collagen cleavage. We studied the effects of oxLDL on the expression of CatK by qRT-PCR and western blot. Gene expression of CatK in oxLDL treated cells was down regulated in a dose dependent manner with at least 3 fold decrease (Fig. 4A). CatK activity was assayed by fluorometric assay using synthetic substrate. The CatK activity of the treated cells followed the same tendency as the gene expression analysis (Fig. 4B). Protein expression of CatK assessed by immunoblot was however higher in oxLDL treated cells compared with control cells (Fig. 4C). We therefore hypothesized that oxLDL inhibits the secretion of CatK which leads to its accumulation. To test this hypothesis, we carried out a gelatin zymography and western blot analysis of conditioned media. Equal volume of conditioned media from control and oxLDL treated cells was loaded for CatK zymography and in parallel for western blot under non-reducing conditions. The zymogram showed 3 active bands at ~75 kDa, 37 kDa and ~26 kDa (Fig. 4D). CatK has been shown to form dimers because the complex unwinding of triple helical collagen is necessary for its cleavage^19^. Here, we show that the active band at ~75 kDa was CatK dimer, and there was a complete inhibition of CatK dimer activity at all oxLDL concentrations tested. The CatK active band at 37 kDa also showed a dose dependent decrease in oxLDL treated cells; there was a 3 fold decrease at 0.43 µM, a 5 fold decrease at 0.86 µM and a 12.5 fold decrease at 1.0 µM respectively.

An interesting observation in the zymogram was an active proteolytic activity at ~26 kDa (Fig. 4D) only in oxLDL treated cells. This band showed a dose dependent increase. We assumed that this was some form of truncated CatK.

**Figure 5:**
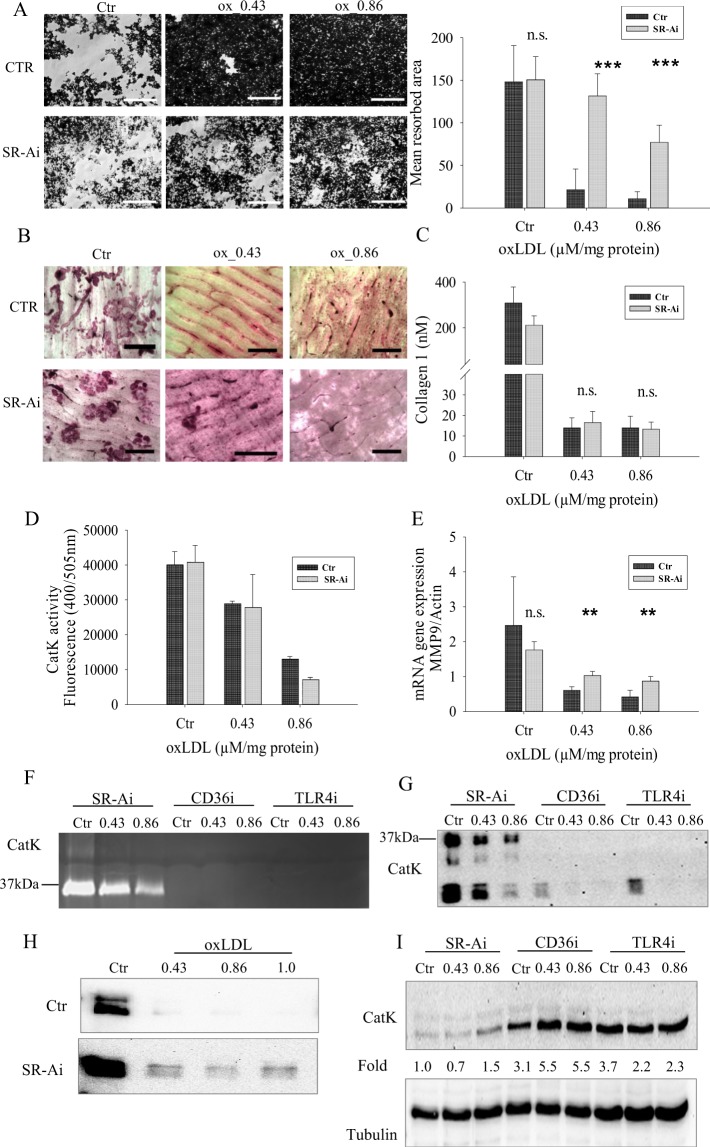
SR-A inhibition rescues some of the oxLDL mediated effects in osteoclasts. (**A**) Increased dissolution of hydroxyapatite coated plates when SR-A is inhibited. Resorptive pits were visualized using a bright field microscope and quantified by ImageJ. Magnification x10 scale bar 1 mm. (**B**) SR-A inhibition did not improve the resorptive activity of oxLDL treated cells on bone slices. Magnification x10, scale bar 1.5 mm. (**C**) Conditioned media from B was collected and analyzed for the presence of collagen type 1 fragment using a colorimetric ELISA method. (**D**) CatK activity by fluorometric assay using synthetic substrate in oxLDL treated cells with and without SR-A inhibition. (**E**) mRNA expression of MMP-9 with/without oxLDL and SR-A inhibition. (**F**) Conditioned media from control and oxLDL treated osteoclasts were subjected to gelatin zymography with SR-A, CD36 and TLR4 inhibitions. (**G**) Conditioned media were subjected to western blot analysis and probed with the CatK antibody after SR-A, CD36 and TLR4 inhibitions. (**H**) Conditioned media from control and oxLDL treated osteoclasts grown on bone slices were subjected to western blot analysis and probed with the CatK antibody. (**I**) Cells were lysed at day 3 and equal amount of cell lysates were western blotted for CatK. Only inhibition of SR-A increased the secretion of CatK, hence decreasing CatK content in cell lysates. Images from both zymography and western blot were captured using the VersaDoc‐3000 (Bio‐Rad Laboratories, Muenchen, Germany).

Likewise, we tested the effects of oxLDL on the expression and secretion of matrix metalloproteinase 9 (MMP-9). MMP-9 is shown to be highly expressed by osteoclasts and could be involved in its migration though its role in bone matrix dissolution remains unclear^11^. As shown in Figs 4F and 5E, there was a down regulation in the gene expression and secretion of MMP9 by oxLDL respectively.

### oxLDL effects are mediated through the scavenger receptor A (SR-A)

Further, we employed specific pharmaceutical inhibitors to decipher the receptor involved in the oxLDL mediated regulation of osteoclastogenesis. We expected a rescue effect leading to an improved functional activity of oxLDL treated cells. Four major receptors have been shown to bind oxLDL; these are the CD36, LOX-1, TLR-4 and SR-A^3^. Inhibition of CD36, LOX-1(data not shown) and TLR-4 receptors did not rescue the oxLDL mediated effects as shown in Fig. 5. The dissolution of calcium crystals in oxLDL treated cells did not increase (data not shown), CatK secretion was still repressed (Fig. 5F,G) leading to an accumulation of CatK as shown by western blot analysis of cell lysate (Fig. 5I). Nevertheless, CD36, TLR-4 and LOX-1 receptors seem to be important for the process of osteoclastogenesis since inhibiting these receptors disturbed the normal functioning of osteoclasts even in the absence of oxLDL. To rule out cytotoxicity of the inhibitors on osteoclast formation and function, we carried out LDH assay. The data shown in the Supplementary Fig. 6 demonstrate that inhibitors used did not cause cell toxicity.

Blocking SR-A with its competitive inhibitor; dextran sulphate^20^, rescued some of the oxLDL mediated effects: there was an increase in the dissolution of calcium crystals (Fig. 5A), an upregulation in the expression of MMP-9 gene (Fig. 5E) and an increased secretion of CatK (Fig. 5F–I). However, it did not increase resorption on bone slices (Fig. 5B and C) and CatK activity (Fig. 5D). We expected that a higher CatK secretion would boost bone resorption in the presence of oxLDL; however, this was not the case and might suggest that catalytically inactive forms of CatK were secreted.

### oxLDL inhibits osteoclastogenesis by inhibiting autophagy

**Figure 6:**
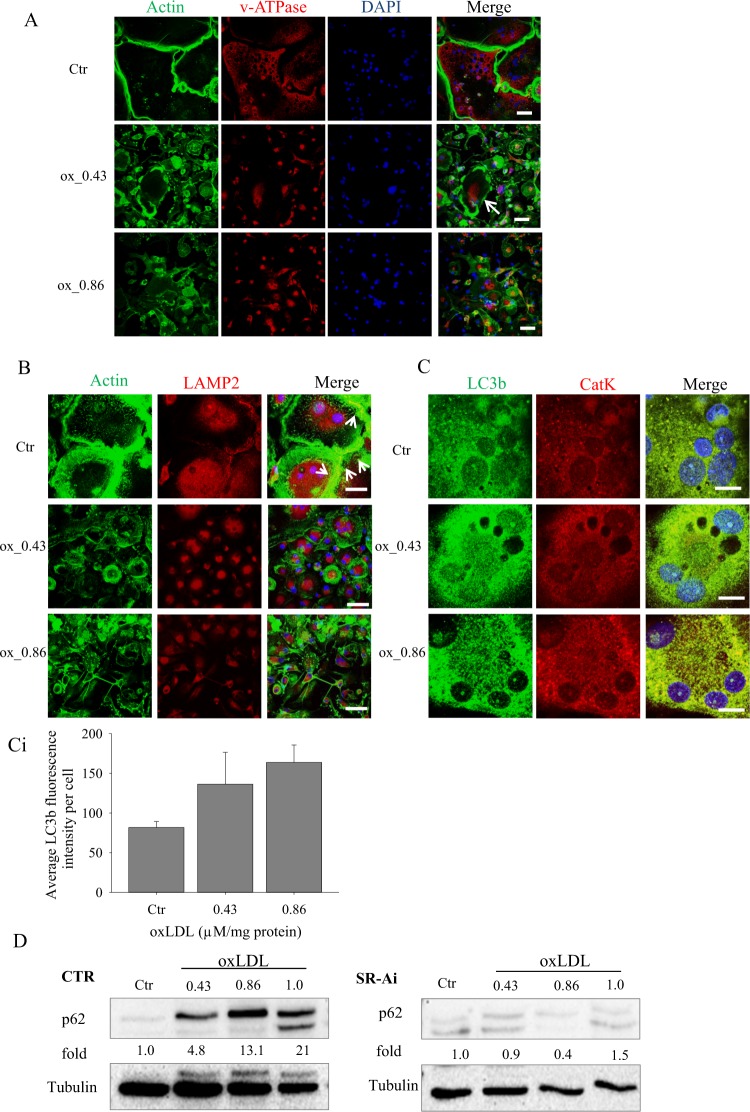
oxLDL inhibits targeting of lysosomal proteins to the ruffled border through autophagy regulation. (**A**) Mature osteoclasts were stained with FITC-phalloidin (green) and co-stained with v-ATPase (subunit a3) (red) Magnification x40, scale bar 50 µm. White arrows indicate cytoplasmic localization of v-ATPase. (**B**) Mature osteoclasts were stained with FITC-phalloidin (green) and then co-stained with the lysosomal protein; LAMP2 (red) Magnification x63 scale bar 50 µm. White arrows indicate peripheral localization of LAMP2. (**C**) Monocytes plated on glass coverslips were grown in the presence of M-CSF and RANKL for a total of 15 days. Mature osteoclasts were stained with the lysosomal marker LC3b (in green) and then co-stained with CatK (in red). Magnification x63, zoom 4x, scale bar 15 µm. Quantification by ImageJ. (**D**) Cells were lysed at day 3 and equal amount of cell lysates were western blotted for p62.

As we have already described above, secretion of lysosome resident protein is important for the functional activities of osteoclasts. One of the cellular mechanisms proposed for secretion of lysosomal proteins in osteoclasts suggests that proteins are packaged in secretory lysosomes that subsequently fuse with the ruffled border and discharge its content to the extracellular space^21^. To address if oxLDL controls targeting of lysosomal vesicles to the ruffled border, we investigated the localization of V-ATPase proton pump (V-a3) in control and oxLDL treated cells. The proton pump isoform V-a3 is highly expressed in osteoclasts and assemble in the plasma membrane of mature osteoclasts^22^. Confocal images showed that the V-a3 proton pump localized at the cell periphery of control cells while it was confined to the cytoplasm of oxLDL treated cells (Fig. 6A), confirming the above indicated acridine orange staining results (Fig. 3C). To further address these effects of oxLDL, we analyzed the localization of the integral lysosomal membrane protein 2 (LAMP2), which resides across lysosomal membranes. Confocal images in control cells showed lysosomal membrane proteins co-localizing with actin rings in the ruffled border thereby implying an active lysosomal fusion with the plasma membrane. In the presence of oxLDL, LAMP2 accumulated mainly in the cytoplasm (Fig. 6B), suggesting an impaired targeting of lysosomal vesicles to the ruffled border.

Studies have described how the fusion of secretory lysosomes to the ruffled border resembles fusion of autophagosomes to lysosomes, indicating that autophagy proteins are important for the secretory function of osteoclasts and participate in the release of lysosome-resident resorptive molecules^23^. With this in mind, we first analyzed the lysosomal marker LC3b co-stained with CatK by confocal microscopy. As depicted in Fig. 6C, there was an increased accumulation of LC3b lysosomal vesicles in oxLDL treated cells compared with control cells suggesting a blockage of autophagy.

To verify if the inhibited targeting of lysosomal vesicles to the ruffled border by oxLDL treated cells and the accumulation of LC3b vesicles was because of a blockage of autophagy, we checked the expression of p62; a protein that has been shown to be degraded by the autophagy-lysosomal pathway. Treatment with oxLDL led to an accumulation of p62 suggesting that oxLDL inhibits the process of autophagy. However, p62 levels became comparable with control cells when SR-A was inhibited (Fig. 6D).

**Figure 7:**
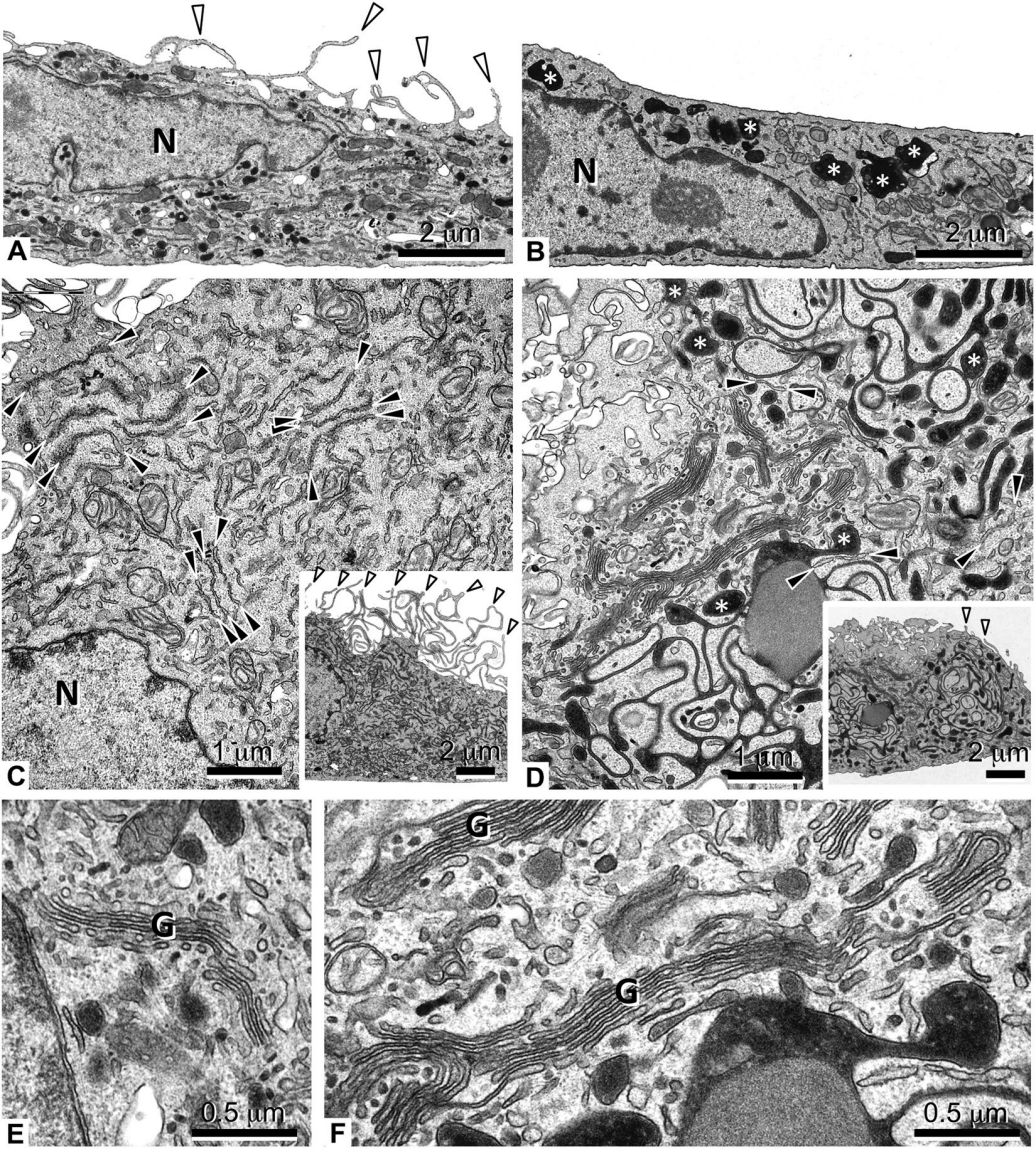
Transmission electron microscopy of osteoclasts. Monocytes were plated in plastic cell culture dishes. Differentiation to osteoclasts and oxLDL treatment was performed as described. Control (**A**,**C**,**E**) and oxLDL treated (**B**,**D**,**F**) treated cells are shown. (**A**,**B**) Overviews showing the apical (top) and basal (bottom) side of the cells. Note the apical cell extensions (white arrowheads) in the control, which are mostly absent in the oxLDL treated. Electron dense, membrane surrounded compartments (**B**, asterisks) were predominantly observed in oxLDL treated osteoclasts. Higher magnifications of the cytoplasm (**C**,**D**; insets: overviews of the cells, white arrowheads: apical cell extensions) reveal rough endoplasmic reticulum (ER) in both samples (**C**,**D**; black arrowheads). In the oxLDL treated cells, electron dense compartments (**D**, asterisks) also appear as cisternae, in that case only few ER cisternae are observed. Golgi stacks appear enlarged in oxLDL treated osteoclasts (**E**,**F**). N, nucleus; (**G**) Golgi. Scale bars are 2 µm in (**A**,**B**) 1 µm in (**C**,**D**) 2 µm in the insets of (**C**,**D**) 0.5 µm in (**E**,**F**) n = 2.

Further, control and oxLDL treated osteoclasts were compared using transmission electron microscopy. The control cells showed cell extensions on the apical membrane in mostly all sections. These were mostly absent or clearly diminished in the osteoclasts treated with oxLDL (Fig. 7A,B, insets in C,D). This observation suggests disturbance of the functional secretory domain of the apical side of the osteoclasts in the presence of oxLDL. Electron dense compartments were clearly visible in oxLDL treated cells, which appear separate, membrane surrounded, irregular shaped (B), or sometimes connected in a syncytium of electron dense cisternae (D). In the latter case, the typical cisternae of rough endoplasmic reticulum (Fig. 7C) are less predominantly observed (Fig. 7D). Further, oxLDL treated cells showed massive enlargement of Golgi stacks compared to the control (Fig. 7E,F). Together, this data point to massive cytoskeletal and vesicles trafficking disturbances and intracellular accumulation of protein-dense vesicles in oxLDL-treated cells.

## Discussion

Osteoclasts play a very crucial role not only for bone remodeling but also for calcium homeostasis^24^. Disturbance of the delicate balance between bone forming cells and bone resorbing cells affects bone health and can cause extra skeletal calcification of soft tissues. Several report show that oxLDL inhibits differentiation of osteoclasts^6^. Whereas, contradictory data are reported regarding oxLDL effects on osteoblasts with some showing inhibition^25^ and others activation^26^ of osteoblasts function. In blood vessel wall, oxLDL is phagocytosed by macrophages and leads to the development of atherosclerotic plaques^27^.

This research work provides a mechanistic insight on the effect of oxLDL to the process of osteoclastogenesis, emphasizing that this compound plays an important role on bone remodeling.

We have shown that oxLDL inhibits osteoclastogenesis. In some studies, the number of TRAP positive cells are used as a readout of successful osteoclastogenesis^6^. Interestingly, we have found that at 0.43 µM oxLDL concentration, there were even slightly higher numbers of TRAP positive cells compared with the control. However, the number of nuclei per cell used as an index for osteoclast activity^28^ was at least 3 fold lower at the same oxLDL concentration. In our view, this means that TRAP staining could be an insufficient measure of osteoclastogenesis. A reason might be that the expression of TRAP is not exclusive to osteoclasts. Several cells such as macrophages, neurons, T-cells have been shown to express TRAP and the mechanisms governing its action is not fully understood^11,29^. In contrast, the number of nuclei per TRAP positive cell mirrors the fusion process and might be the better read-out for a successful osteoclastogenesis.

We have also identified SR-A as an important receptor mediating oxLDL effects in osteoclast precursor cells. SR-A is a pattern recognition receptor expressed mainly by macrophages. It has a broad ligand binding ability suggesting diverse roles ranging from cell adhesion to pathogen clearance to cytokine production and to signal transduction^30,31^. Lin *et al*.^32^, explained the importance of SR-A in bone development *in vivo* by deletion of SR-A which resulted in a decreased number of osteoclasts leading to a higher bone mass in knockout mice. Takemura *et al*.^33^, also reported the expression of SR-A during the different stages of osteoclastogenesis, emphasizing its importance in bone biology. However, the role of SR-A in the presence of endogenous compounds such as oxLDL on osteoclast precursor cells has not yet been explored. This study therefore demonstrate that SR-A mediates the recognition of oxLDL in osteoclast precursor cells and its inhibition rescues some of the oxLDL mediated effects on osteoclastogenesis.

Studies by Takemura *et al*.^33^, showed a decrease in the expression of SR-A at a later time point of osteoclastogenesis, indicating that mature osteoclasts possibly have no expression of SR-A. We suppose that the reversal of some of the oxLDL mediated effects by SR-A were successful because they took place early at osteoclastogenesis. This could explain why some of the oxLDL mediated effects such as resorption on bone slices could not be reversed when SR-A was inhibited, since a longer incubation time is needed and SR-A expression might have decreased. Therefore, further studies are needed to identify which receptors and pathways are activated by osteoclasts when SR-A expression decreases. The other 3 pattern recognition receptors; CD36, TLR-4 and LOX-1 also known to bind oxLDL^3^ are important for osteoclastogenesis, since blocking them repress the process of osteoclastogenesis as evidenced in the suppression of CatK secretion and accumulation of CatK in the cell.

The polarization of osteoclasts differentiates between an active and a non-active osteoclast and the formation of actin rings indicates the status of polarization as well as cell activity^34^. oxLDL interferes with this process leading to inactive osteoclasts that lack the ability to reorganize and form sealing zones that shield the resorption site from the environment. At first we thought oxLDL inhibits the expression of integrins which disable the cell matrix adhesion leading to a disruption in the cytoskeletal rearrangements. We however observed that the expression of integrin in oxLDL treated osteoclast was similar to control (Supplementary Fig. 1). Studies by Feng *et al*.^22^, described the importance of V-a3 proton pump localization and its stabilization in the formation of actin rings. They also reported that a depletion of the V-a3 isoform results in a defective actin ring formation in mature osteoclasts. As described above, oxLDL inhibited the localization of V-a3 isoform to the cell periphery suggesting that the inefficient delivery of V-a3 to the ruffled border in oxLDL treated cells could directly affect cytoskeletal rearrangements in osteoclasts.

One major function of osteoclasts is the dissolution of bone matrix, and we show for the first time a direct correlation between CatK secretion and the resorptive activity of osteoclasts on hydroxyapatite coated plates. Secretory cells such as osteoclasts exhibit active machinery that secrets lysosomal enzymes to the extracellular space for bone degradation, and CatK has been identified to be a crucial protease for bone resorption. We illustrate that oxLDL repressed the secretion of CatK and the resorptive activity on hydroxyapatite coated plates. This was directly correlated with the secretion and resorptive pattern on bone slices which could be due to the off-target localization of lysosomal proteins. Although there is a direct correlation between CatK secretion and osteoclastic resorption on hydroxyapatite coated plates, it is well known that CatK is a protease that degrades organic bone matrix (collagen) and not inorganic bone components (calcium phosphate). However, our data suggests that hydroxyapatite coated plates could also suffice in stimulating CatK secretion since similar pattern of CatK release was observed in both cells grown on plates and bone slices.

Previous work published by Cremasco *et al*.^35^, demonstrate different pathways for cathepsin K exocytosis and proton pump exocytosis in osteoclasts. They noted that while osteoclasts from PKCδ deficient mice could dissolve hydroxyapatite coated plates, CatK secretion important for organic bone degradation was inhibited. Our study clearly shows that oxLDL repressed the lysosomal exocytosis of both V-a3 and CatK. This was evident in the inefficient delivery of the proton pump to the ruffled border leading to an inhibition of dissolution of hydroxyapatite coated plates and in reduced CatK secretion respectively. Our findings further suggest that the identified mechanism disturbed by oxLDL during osteoclastogenesis is the process of autophagy, resulting in the intracellular accumulation of LC3b^+^ lysosomal vesicles. Although, oxLDL does not hinder the packaging of cargo into lysosomal vesicles which has been evident in the accumulation of LC3b/CatK positive vesicles in oxLDL treated cells, the fusion of these vesicles to the ruffled border is inhibited. This could be due to the disruption of the cytoskeletal rearrangement (Fig. 2) as a result of the off-target localization of the proton pump in the cytoplasm (Fig. 6) so that proteins in lysosomal vesicles could not be directed to the ruffled border.

**Figure 8:**
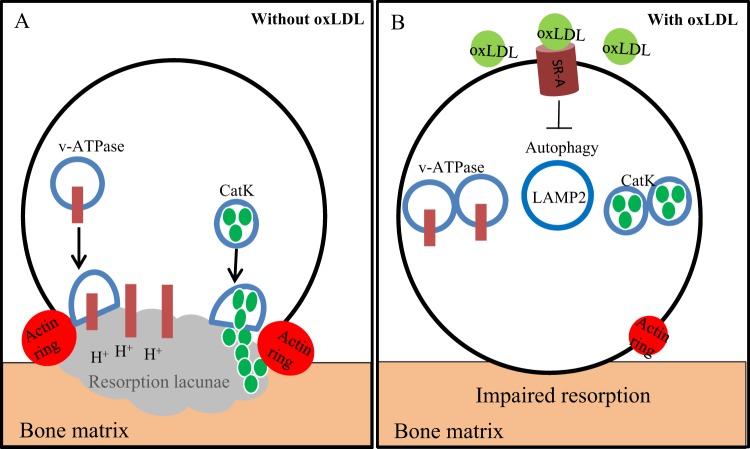
Regulation of CatK secretion by oxLDL. (**A**) The process of osteoclastogenesis without oxLDL. LAMP2 residing vesicles (v-ATPase and CatK) fuse readily to the ruffled border leading to an active protonation of the resorption lacunae and CatK secretion. Cytoskeletal rearrangement leads to actin rings which actively seals the resorption site from the environment. (**B**) The process of osteoclastogenesis when oxLDL is present. oxLDL binds to the SR-A in osteoclast precursor cells inhibiting the process of autophagy. This leads to an accumulation of LAMP2 residing vesicles (v-ATPase and CatK) in the cell and hence, an impaired bone resorption. Also the cytoskeletal rearrangement is impaired leading to actin rings that cannot shield the resorption site from the environment.

Transmission electron microscopy study further confirmed a general disturbance of vesicle trafficking in oxLDL treated osteoclasts. Thus, we observed dramatic decrease in the number of membrane protrusions on the apical side of osteoclasts (Fig. 7). This suggests that functional secretory domain is also disturbed by oxLDL. Functional secretory domain is involved in transcytotic transfer of type I collagen fragments and other factors released from the bone during resorption such as TGF-β and undercarboxilated osteocalcin^36^. This data confirms results presented in Fig. 3D,E showing diminished release of collagen fragments in the presence of oxLDL. Furthermore, oxLDL treated cells showed accumulation of electron dense structures most likely representing protein-loaded vesicles. Golgi hypertrophy has also been documented to be associated with lysosomal accumulation of oxLDL in macrophages^37^. It remains to be investigated to what extent osteoclasts can accumulate oxLDL intracellularly and how this affects cell function. Our data are summarized schematically in Fig. 8.

In conclusion, the data we present here show the importance of SR-A in mediating the uptake of oxLDL in osteoclast precursor cells and its effects on osteoclastogenesis. More importantly, we show that oxLDL does not affect the packaging of cargo into the lysosome but restrain the fusion of lysosomal vesicles to the ruffled border by inhibiting the process of autophagy.

## Materials and Methods

### Cell culture and osteoclastogenic differentiation

Peripheral blood mononuclear cells (PBMCs) were isolated from buffy coats (DRK-Blutspendedienst NSTOB, Springe, Germany. All methods were carried out in accordance with relevant guidelines and regulations. All experimental protocols were approved by the Ethics Committee of the Hannover Medical School. All donors signed the informed consent) with the lymphocyte separation medium (PAA Laboratories, Coelbe, Germany) according to standard protocol. After density gradient centrifugation, monocytes were isolated by depletion of non-monocytes using the monocyte isolation kit (Militenyi Biotec, Bergisch Gladbach, Germany) according to manufacturer’s instructions. Monocytes were plated at a density of 2 × 10^5^ cells per well in 96 well plates, hydroxyapatite coated plates and bone slices, 5 × 10^5^ cells per well on glass coverslips in 24 well plates and 1 × 10^6^ in 6 well plates. Cells were cultured in Alphamem (Biochrom, Berlin, Germany) medium supplemented with 10% fetal calf serum (FCS) and 20 ng/mL M-CSF (Peprotech, New-Jersey, USA) for 7 days for differentiation into macrophages; medium was changed at day 3 during this period. For differentiation of the macrophages to osteoclasts, cells were grown in osteoclastogenic medium consisting of 20 ng/mL M-CSF and 20 ng/mL RANKL (Peprotech, New-Jersey, USA) for 8–14 days with medium changed every 4 days.

### oxLDL preparation

LDL was isolated from human plasma obtained from DRK-Blutspendedienst NSTOB, Springe, Germany (All methods were carried out in accordance with relevant guidelines and regulations. All experimental protocols were approved by the Ethics Committee of the Hannover Medical School. All donors signed the informed consent) and oxidized with CuSO_4_ as previously described^38,39^. oxLDL was characterized by the protein concentration measured by DC-protein assay (Biorad, Munchen, Germany) and lipid peroxidation measured by the TBARS assay (Cayman chemicals, Michigan, USA). oxLDL concentration of 0.43 µM/mg protein (~10 ug/mL protein), 0.86 µM/mg protein (~20 ug/mL protein) and 1 µM/mg protein (~25 ug/mL protein) was used. Stimulation of the osteoclast with oxLDL and inhibition of the several receptors (CD36, LOX-1, TLR-4 and SRA-1) were all done at the beginning of osteoclast differentiation i.e. when RANKL was added. CD36, LOX-1, TLR-4 and SR-A were inhibited by 10 µM of sulfosuccinyloleate (SSO) (Cayman chemicals, Michigan, USA), 250 µM of κ-Carrageenan (Sigma, Steinheim, Germany), 5 µM of CLI-095 (Invivo-Gen) and 100 µg/mL of dextran sulphate (Sigma, Steinheim, Germany) respectively.

### Preparation of hydroxyapatite coated plates

Hydroxyapatite coated plates were prepared as described by Maria *et al*.^40^, with slight modifications. Briefly, culture plates were incubated twice with 400 µL simulated body fluid (SBF; 50% Tris buffer (50 mM Tris base, pH 7.4 with 1 M HC1), 25% calcium stock solution (25 mM CaCl_2_.H_2_O, 1.37 M NaCl, 15 mM MgCl_2_.6H_2_O in Tris buffer, pH 7.4), and 25% phosphate stock solution (11.1 mM Na_2_HPO_4_.H_2_O, 42 mM NaHCO_3_ in Tris buffer, pH 7.4) for 3 days each at room temperature. SBF was aspirated and 400 µL of calcium phosphate solution (CPS; 41 mM HCl, 2.25 mM Na_2_HPO_4_.H_2_O, 4 mM CaCl_2_.2H_2_O, 0.14 M NaCl, and 50 mM Tris, pH to 7.4) was also added twice for 1 day at room temperature. Cell culture plates were then washed with 70% ethanol for sterilization. Prior to cell plating, these hydroxyapatite coated plates were activated with fetal calf serum (FCS) for 1 hour at 37 °C to increase cell adherence.

### Characterization of osteoclastic activity on hydroxyapatite coated plates

After culturing the cells on the hydroxyapatite coated plates for a total of 3 weeks; with or without oxLDL and with or without receptor inhibition, cells were removed by addition of 200 µL of 1 M NaCl in 0.2% Triton X-100 solution for 2 minutes. The plates were then washed twice with distilled water, air dried and viewed under a bright field microscope. Images were taken and resorption areas measured by ImageJ.

### Osteoclast acidification assay

Intracellular acidification was determined by acridine orange (Cayman chemicals, Michigan, USA). Thirteen micrograms of acridine orange were incubated with cells in culture medium for 45 minutes. Cells were washed twice with PBS and processed for fluorescence microscopy using the Leica DMI3000 B microscope at 485 nm excitation and 520 nm emission.

### TRAP activity staining

The cells were fixed and stained with acid phosphatase leukocyte (TRAP) kit (Sigma-Aldrich, Steinheim, Germany) according to manufacturer’s instructions after culturing for a total of 21days with and without oxLDL and with or without receptor inhibition.

### PIT assay

Cells were cultured on bovine cortical bone slices (Bone slices.com, Jelling, Denmark) as earlier described. After the stipulated time point, cells were removed by incubating for 10 minutes with 5% sodium hypochlorite, washed twice with PBS and then stained with 1% toluidine blue (Sigma, Missouri,USA) (1% toluidine blue dissolved in 1% sodium tetraborate decahydrate). Cortical bone slices were then washed in tap water, air dried and viewed under the bright field microscope. Culture supernatant was collected at different time points and assayed for collagen type 1 fragment using the CrossLaps ELISA (Immunodiagnostics, Frankfurt, Germany) according to manufacturer’s instructions.

### Gelatin Zymography

Cathepsin zymography was performed as previously described^41^. Briefly, 6X non reducing loading buffer (0.05% bromophenol blue, 0.5 M Tris HCl, 50% glycerol, 10% SDS) was added to all samples prior to loading. Samples were then resolved by 12.5% SDS-PAGE containing 0.2% gelatin at 4 °C (30 mA/gel, 0.75 mm thick minigel). The gels were carefully removed and enzymes renatured 3X for 10 minutes in renaturing buffer (65 mM Tris buffer, pH 7.4 with 20% glycerol). Afterwards, gels were incubated in activity buffer (0.1 M phosphate buffer, 1 mM EDTA and freshly added 2 mM DTT pH 6.0) for 30 minutes at room temperature and then changed to a fresh activity buffer and incubated at 37 °C for 72 hours. Gels were then washed twice with distilled water and then stained for 30 minutes in coomassie stain (0.5% coomassie (Sigma, Steinheim, Germany) in 30% ethanol and 10% acetic acid). Gels were then destained in destaining solution(30% ethanol and 10% acetic acid) until clear bands appeared. To also detect secreted CatK by western blot, SDS page was carried out as outlined above without gelatin substrate polymerized in the resolving gel. Protein sample was transferred to a PVDF membrane and probed with the CatK antibody (dilution 1:500, SantaCruz biotechnology, Heidelberg, Germany).

### Western blot

After stimulation with RANKL for 3 days with/without oxLDL and receptor inhibition, cells were lysed and processed for western blot as we described previously^42^. The membrane was probed with cathepsinK, MMP9, p62 antibody (SantaCruz biotechnology, Heidelberg, Germany), and images were captured by the VersaDoc-3000 and quantified using the Quantityone software (Biorad laboratories, Muenchen, Germany).

### Gene expression analysis

Cells were cultured with or without oxLDL for the stipulated time. Total RNA was extracted with the RNeasy Miniprep kit (Qiagen, Hilden, Germany) according to manufacturer’s instructions and qRT-PCR was performed on a Light-Cycler 480 real time PCR system with the following primers:

CatK^43^: 5′-ATATGTGGGACAGGAAGAGAGTTGT-3′ (sense), 5′-GGATCTCTCTGTACCCTCTGCATTTA-3′ (antisense), 6-FAM-TGTACAACCCAACAGG CAAGGCAGC-TAMRA.

VNR: 5′-AACTCAAGCAAAAGGGAGCA-3′ (sense), 5′-GGGTTGCAAGCCTGTTGTAT-3′ (antisense), 6-FAM-CTGCGGGATGAATCTGAATT-TAMRA (probe).

GUSB: 5′‐TGGTGCTGAGGATTGGCA-3′ (sense) 5′ TAGCGTGTCGACCCCATTC-3′ (antisense), 6‐FAM‐TGCCCATTCCTATGCCATCGTGTG‐TAMRA (probe). Real-time PCR was also performed using Applied Biosystems Taqman gene expression assays for CalcR (Hs01016882_m1), MMP9 (Hs00957562_m1) and beta actin (Hs01060665).

### Confocal immunofluorescence

After the stipulated time, cells grown on coverslips were fixed and processed for immunostaining as we have previously described^44^. Briefly, cells were stained for alpha-actin with alexa fluor 488 phalloidin (Molecular probes, Goettingen, Germany) and subsequently for CatK (SantaCruz biotechnology, Heidelberg, Germany) LAMP2 (BD biosciences, Heidelberg, Germany), LC3b (Novus Biologicals, Germany), v-ATPase a3 (Thermofisher, Rockford, USA), Vinculin (Chemicon Europe, Southampton, UK) at 4 °C overnight. The corresponding secondary antibody conjugated with alexa fluor 488, alexa fluor 633 and alexa flour 594 for 1 hour at room temperature. DraQ5 (Biostatus Limited, United Kingdom) and DAPI were applied for nuclear staining. For negative controls, samples were incubated with rabbit IgG. Cells were then mounted with Aqua poly mount (Polysciences, Eppelheim, Germany) and analyzed on a Leica TCS-SP2 AOBS confocal microscope (Leica Microsystems). All the images were taken with oil-immersed x40 objective, NA 1.25 and x63 objective, NA 1.4.

### Transmission electron microscopy

If not otherwise mentioned, incubations were at RT. Samples were fixed in the culture dishes 30 min in 150 mM HEPES, pH 7.35, containing 1.5% formaldehyde and 1.5% glutaraldehyde. After immobilization in 2% agarose, samples were incubated 2 hr in an aqueous solution of 1% OsO_4_ containing 1.5% hexacyanoferrat II, washed in water and stored in 1% aqueous uranyl acetate overnight at 4 °C. After washing in water and dehydration in acetone, samples were embedded in Epon. 60 nm ultrathin sections were mounted on formvar-coated copper grids, poststained with uranyl acetate and lead citrate^45^ and observed in a Morgagni TEM (FEI). Images were recorded using a side mounted Veleta CCD camera. Two independent sets of osteoclasts differentiation were used for transmission electron microscopy experiments. Both sets have shown the same results.

### Statistical analysis

All data were analyzed with SigmaPlot software (Systat software, San Jose, CA) using the one-way anova test unless otherwise indicated. Error bars represent standard deviation. *p < 0.05, **p < 0.01, and ***p < 0.001. Unless otherwise stated, n ≥ 3.

### Data availability

All data generated or analyzed during this study are included in this published article (and its Supplementary Information files).

## Electronic supplementary material

Supplementary dataset 1
