# Supplementary material for: oxLDL inhibits differentiation and functional activity of osteoclasts via scavenger receptor-A mediated autophagy and cathepsin K secretion
Source: Sci Rep. 2018 Aug 2;8:11604. doi: 10.1038/s41598-018-29963-w (PMC6072764; doi:10.1038/s41598-018-29963-w)
Supplement: Supplementary file 1 — Supplementary dataset 1 [file 41598_2018_29963_MOESM1_ESM.pdf]

oxLDL inhibits differentiation and functional activity of osteoclasts via scavenger receptor-A mediated autophagy and cathepsin K secretion.

SUPPLEMENTARY DATA

Damilola Dawodu, [1] Margret Patecki, [1] Jan Hegermann[2], Inna Dumler, [1] Hermann Haller[1] and Yulia Kiyan. [1]

[1] Department of Nephrology and Hypertensiology. Hannover Medical School, Hannover, Germany.

[2]Research Core Unit Electron Microscopy, Hannover Medical School , Hannover, Germany.

Address correspondence to: Yulia Kiyan, PhD, Department of Nephrology and Hypertensiology. Hannover Medical School, Carl-Neuberg-Str. 1, D-30625 Hannover, Germany.

E-mail: kiian.ioulia@mh-hannover.de

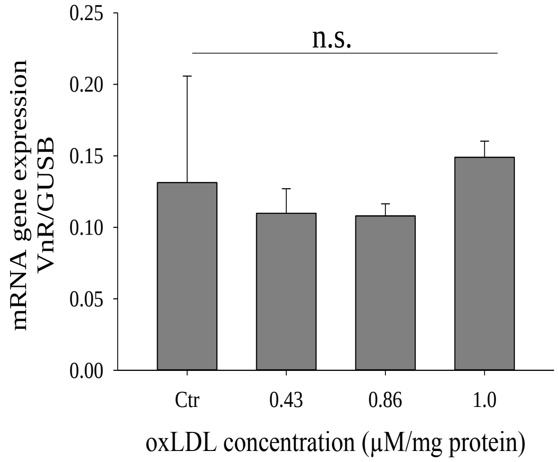

Supplementary Fig 1: oxLDL does not regulate the gene expression of vitronectin receptor; αVβ3 (VnR). mRNA expression of VnR was analyzed in control and stimulated with different concentrations of oxLDL osteoclasts by TaqMan RT-PCR.

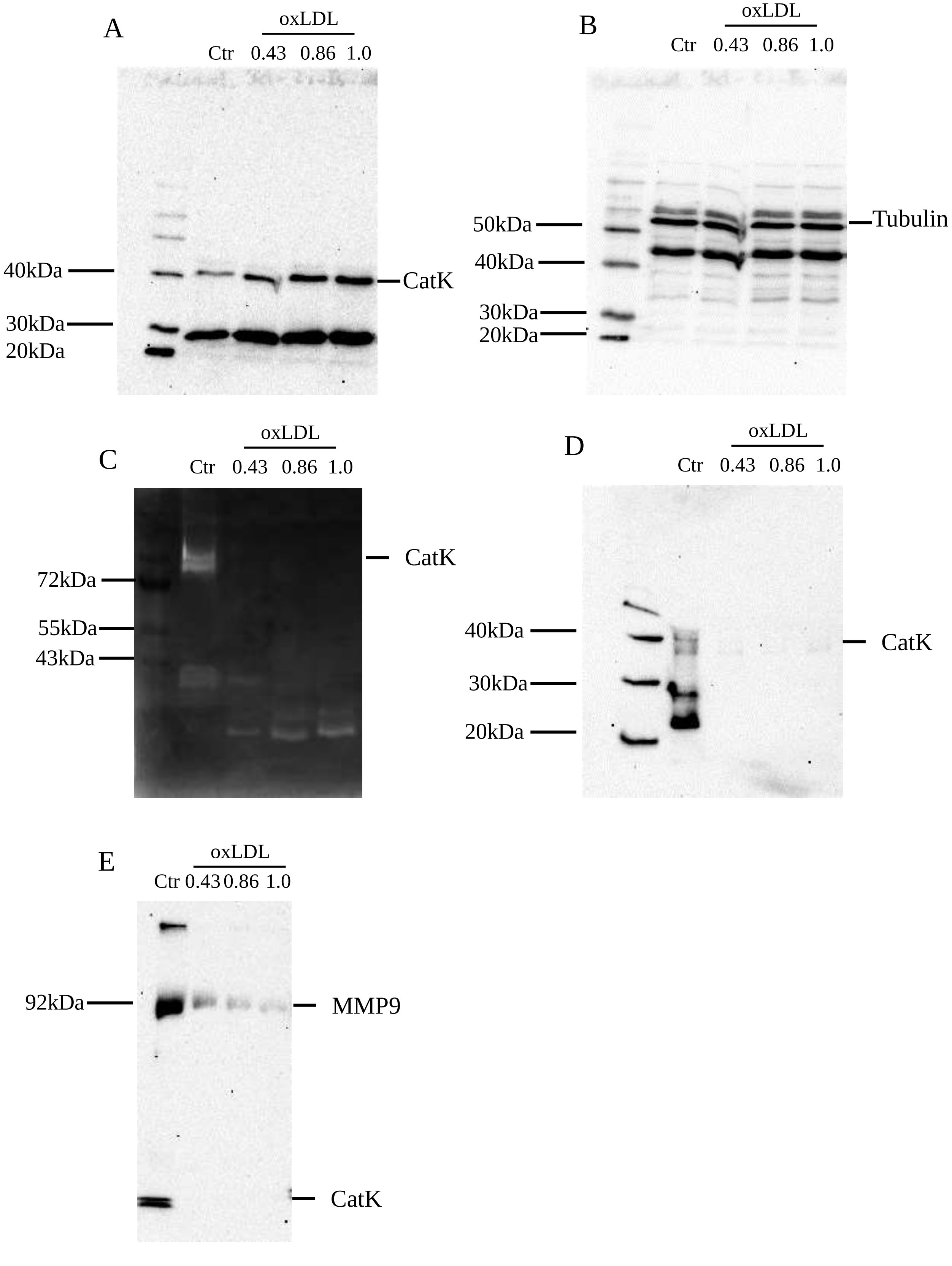

Supplementary Fig. 2: (A): Full length western blot image of CatK shown in figure 4C. (B) Full length western blot image of tubulin shown in figure 4C. (C) Full length gel image of CatK zymography shown in figure 4D. (D) Full length western blot image of CatK shown in figure 4E. (E) Full length western blot image of MMP9 shown in figure 4F.

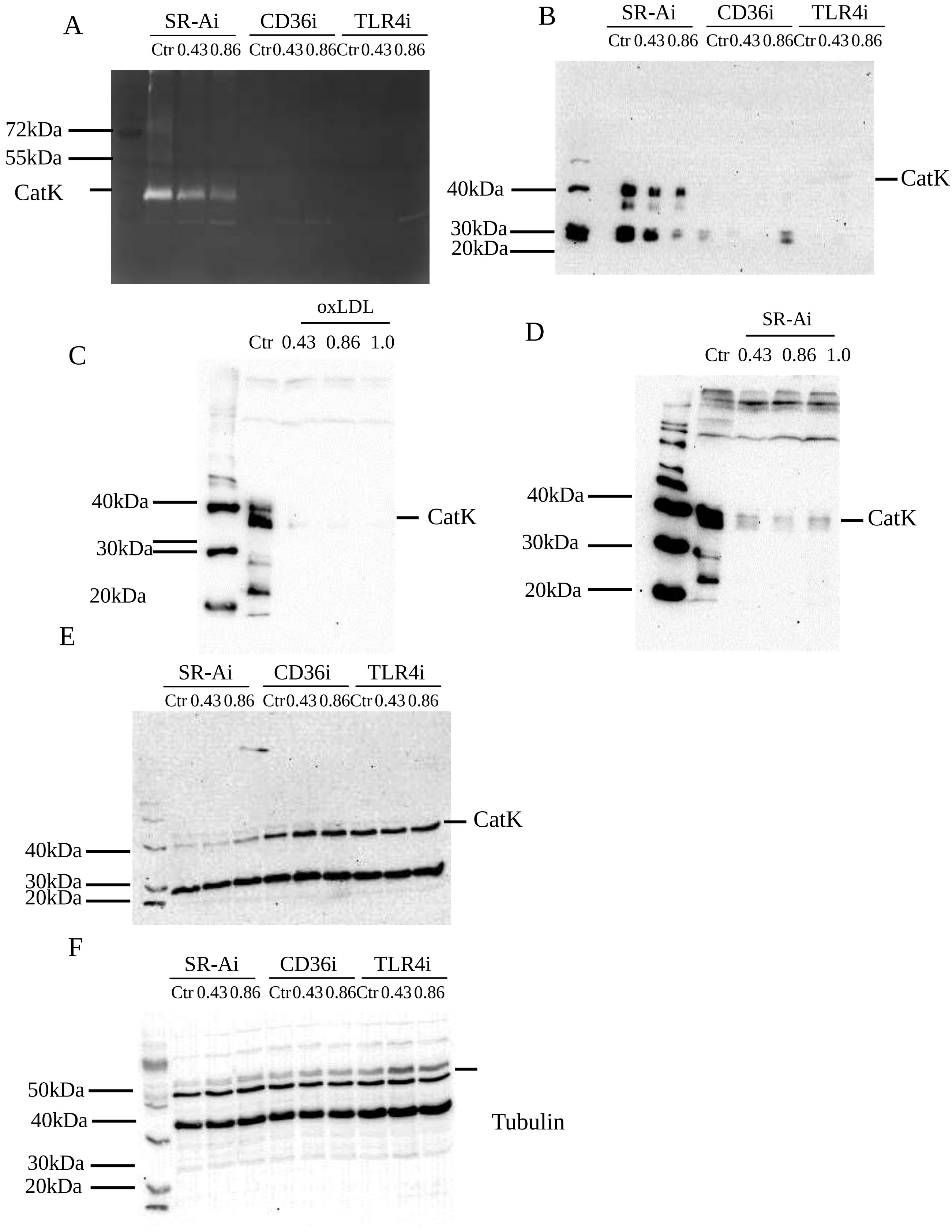

Supplementary Fig. 3: (A): Full length gel image of CatK zymography shown in figure 5E. (B) Full length western blot image of CatK shown in figure 5F. (C) Full length western blot image of CatK shown in figure 5G. (D) Full length western blot image of CatK in the presence of SR-A inhibitor shown in figure 5G. (E) Full length western blot image of CatK shown in figure 5H. (F) Full length western blot image of tubulin shown in figure 5H.

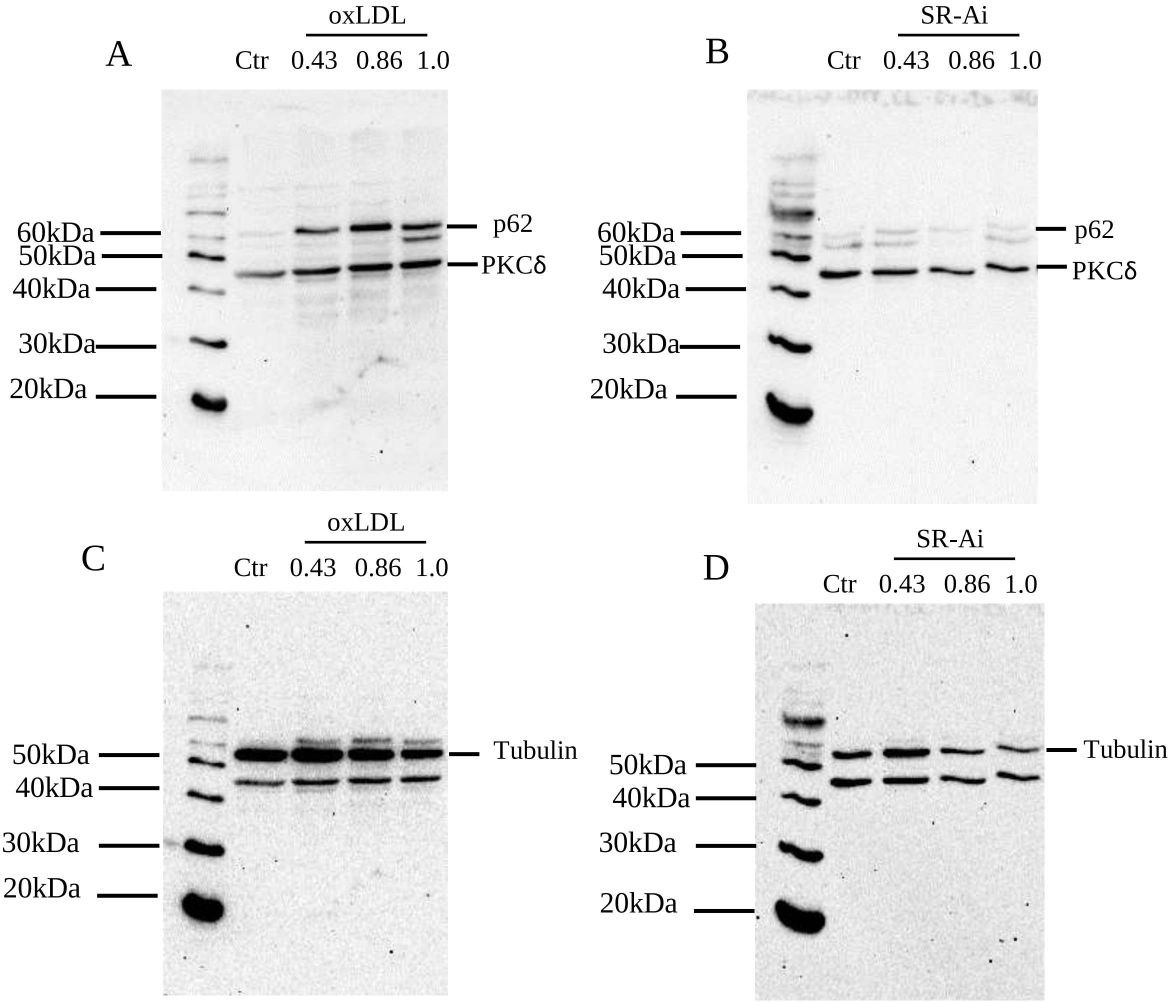

Supplementary Fig. 4: (A): Full length western blot image of p62 shown in figure 6D. (B) Full length western blot image of p62 in the presence of SR-A inhibitor shown in figure 6D. (C) Full length western blot image of tubulin shown in figure 6D. (D) Full length western blot image of tubulin in the presence of SR-A inhibitor shown in figure 6D.

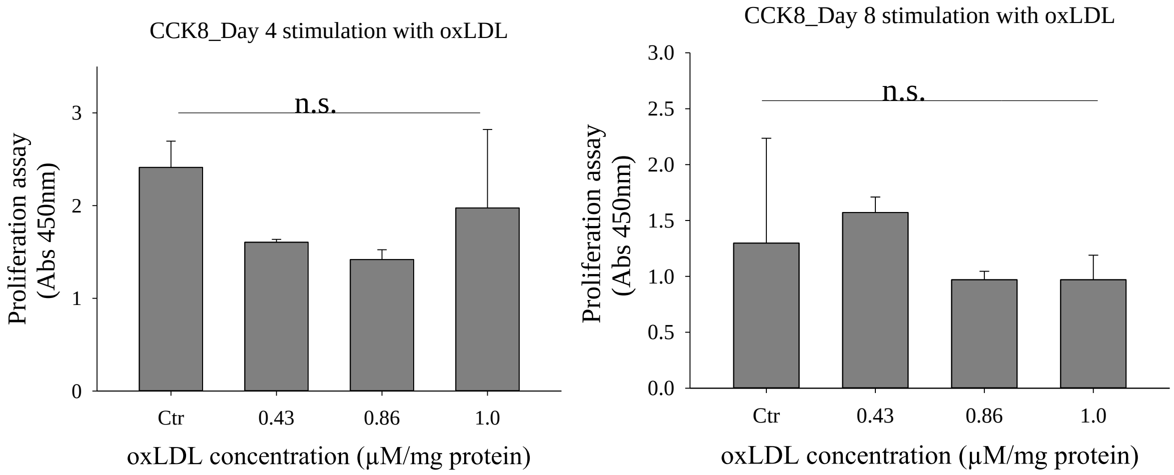

Supplementary Fig. 5: Cells viability of control and oxLDL treated osteoclasts after 4 and 8 days was assessed using CCK8 Cell Counting Kit.

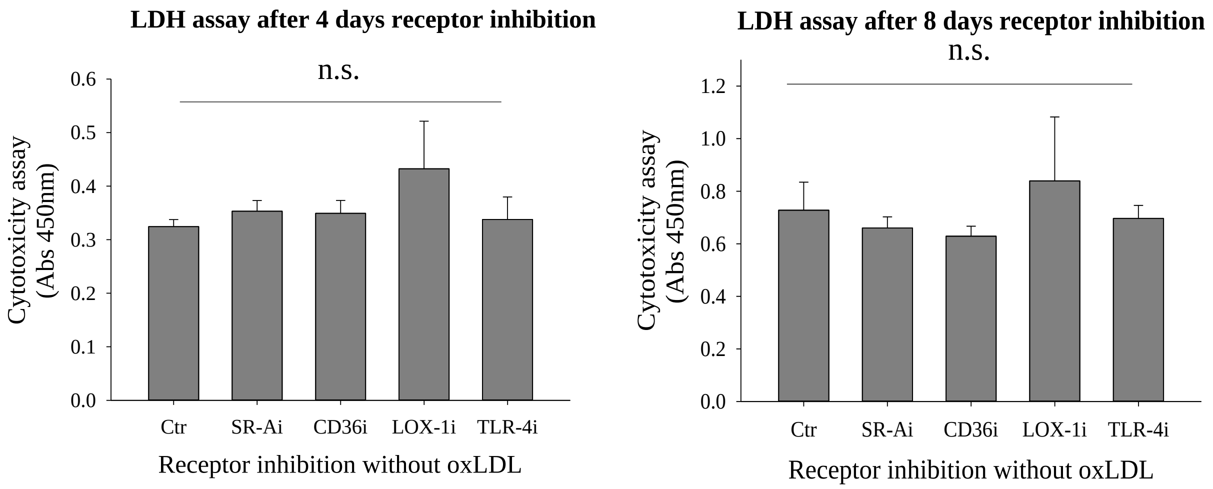

Supplementary Fig. 6: LDH assay of osteoclasts treated with different receptor inhibitors after 4 and 8 days.
